# Determinants of COVID-19 vaccine hesitancy among health care workers in Amhara region referral hospitals, Northwest Ethiopia: a cross-sectional study

**DOI:** 10.1017/S0950268821002259

**Published:** 2021-10-14

**Authors:** Agazhe Aemro, Nakachew Sewnet Amare, Belayneh Shetie, Basazinew Chekol, Mulugeta Wassie

**Affiliations:** 1 Department of Medical nursing, School of Nursing, College of Medicine and Health Science, University of Gondar, Gondar, Ethiopia; 2 Department of Midwifery, Debre Berhan University, Debre Berhan, Ethiopia; 3 Department of Emergency and Critical Care Nursing, School of Nursing, College of Medicine and Health Science, University of Gondar, Gondar, Ethiopia; 4 Department of Anesthesiology, College of Medicine and Health Science, Debre Tabor University, Debre Tabor, Ethiopia

**Keywords:** COVID-19, Ethiopia, healthcare workers, hesitancy, vaccine

## Abstract

Vaccine hesitancy remains a serious global threat to achieve herd immunity, and this study aimed to assess the magnitude and associated factors of coronavirus disease-19 (COVID-19) vaccine hesitancy among healthcare workers (HCWs) in Amhara regional referral hospitals. A web-based anonymised survey was conducted among 440 HCWs in the Amhara region referral hospitals. The questionnaire was designed using Google Forms and distributed using telegram and e-mail from 15 May to 10 June 2021 to the randomly selected participants in each hospital. The data were analysed with Stata 14.0 and described using frequency tables. A multivariable binary logistic regression model was fitted and model fitness was checked with the Hosmer–Lemeshow goodness of fit test. Out of 440 participants, 418 were willing to participate in the study and the mean age was about 30 years. Overall, 45.9% (*n* = 192) of participants reported vaccine hesitancy. After applying multivariate analysis, age ≤25 years (adjusted odds ratio (aOR) = 5.6); do not wear a mask (aOR = 2.4); not compliance with physical distancing (aOR = 3.6); unclear information by public health authorities (aOR = 2.5); low risk of getting COVID-19 infection (aOR = 2.8); and not sure about the tolerability of the vaccine (aOR = 3.76) were associated with COVID-19 vaccine hesitancy. A considerable proportion of HCWs were hesitant towards COVID-19 vaccine, and this can be tackled with the provision of clear information about the vaccine.

## Introduction

Due to occupational exposure, healthcare workers (HCWs) are prioritised for early coronavirus disease-19 (COVID-19) vaccination [1, 2]. Additionally, the general population assumed that HCWs would have no hesitancy to take the COVID-19 vaccine and expected them to be role models in vaccination programmes [3–9]. However, different studies among HCWs showed that, a considerable proportion of HCWs were hesitant against COVID-19 vaccination.

Studies conducted in different European countries, including UK, Germany, Italy, Greek and France, revealed the magnitude of COVID-19 vaccine hesitancy in HCWs ranged from 8.3% to 28.4% [3, 10–14]. Similarly, vaccine hesitancy among HCWs in Vietnam and Saudi Arabia was 23.9% and 49.5%, respectively [15, 16]. Another two studies conducted in Egypt and Democratic Republic of Congo showed vaccine hesitancy among HCWs was 79% and 72.3%, respectively [8, 17]. Furthermore, COVID-19 vaccine hesitancy was significantly associated with having a younger age, female sex, ethnic group, having negative attitude towards COVID-19 and its preventive measures, non-reliable information from public health authorities, perceiving low risk for infection and vaccine safety concern [3, 10, 11, 13, 14, 16–18].

Now a days, COVID-19 infections are increasing in Ethiopia and, shockingly, daily reports of new infections reach beyond 1000, but the vaccination coverage is inadequate [19, 20]. Even though there are many factors which contributed to the decreased vaccination coverage against COVID-19, hesitancy to take the available vaccine is still a problem in Ethiopia. A study conducted among the general population in Ethiopia showed the magnitude of vaccine hesitancy was 68.8% [4]. However, little is known about COVID-19 vaccination hesitancy among HCWs in Ethiopia. Similarly, understanding hesitancy status and its major contributing factors among HCWs would contribute to the development of efficient COVID-19 vaccination promotion strategies. Thus, this study aimed to assess the magnitude and associated factors of COVID-19 vaccine hesitancy among HCWs in Amhara regional referral hospitals.

## Method and materials

### Study setting

The study was conducted at referral hospitals in the Amhara regional state. According to the Amhara National Regional Health Bureau, Annual Performance Report, the region has 81 hospitals, 858 health centres and 3560 health posts. Among those 81 hospitals in the region, the University of Gondar, Dessie, Felege-Hiwot, Tibebe-Ghion, Debre-Markos, Waldiya, Debre Tabor and Debebirhan are referral hospitals. The number of HCWs in these hospitals was estimated to be 4002 [21, 22].

### Study participants and survey design

The authors applied a cross-sectional, web-based anonymous survey using an online questionnaire. The survey was conducted from May 15 to 10 June 2021. The authors used telegram and email (the most popular social media platforms in Ethiopia) to advertise and circulate the survey link to the participants. Data collectors at each hospital were asked to distribute the survey link to their randomly selected contacts in each hospital. The participants were informed that their participation was voluntary, and consent was implied through their completion of the questionnaire. The inclusion criteria were the respondents who were working during the data collection period.

### Sample size determination

The sample size was determined by using the single population proportion formula by taking the proportion of hesitancy to the COVID-19 vaccine at 22.5%, 95% confidence interval (CI) and 4% marginal error. After adding a 5% non-response rate, the final sample size was 440.

### Sampling technique and procedure

**Fig. 1.**
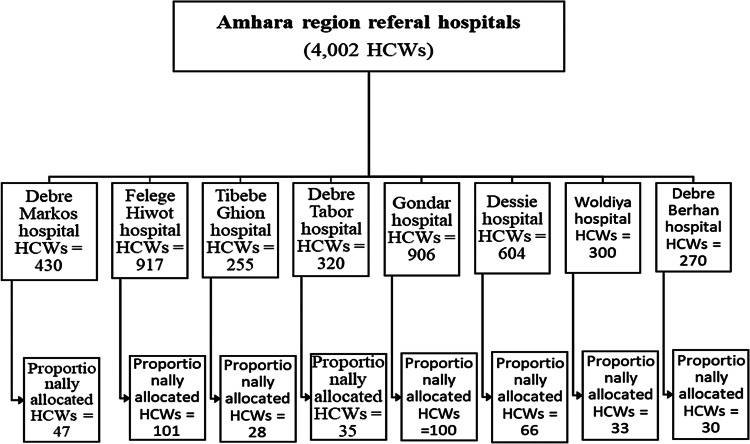
Schematic presentation of the sampling procedure in Amhara region referral hospitals, Ethiopia, 2021.

As shown below in the figure (Fig. 1), the total sample size was first allocated proportionally to those eight hospitals. Then, the link for the questionnaire was given to the data collectors and forwarded it to randomly selected HCWs of each hospital, using e-mail or telegram. The link was forwarded to each hospital's data collector to avoid coverage bias and to be a representative sample.

### Operational definitions

HCW: Any member of the health care unit that includes medical doctors, pharmacists, physiotherapists, midwifery, laboratory technologists, nursing professions or any other person in the course of his or her professional activities who may prescribe, administer or dispense a medicinal product to an end-user [23].

Vaccine hesitancy: World Health Organization (WHO) declared vaccine hesitancy as ‘the reluctance or refusal to vaccinate despite the availability of vaccines’ [24].

COVID-19 preventive behaviours: Refers to hand washing, physical distancing, social isolation and face-mask wearing practices to prevent COVID-19 infection and measured ‘Yes’ or ‘No’ answers to the questions.

Perceived susceptibility/risk of getting COVID infection: Refers to a participant's subjective perception of the risk of acquiring COVID-19 and is measured as High, Moderate, Low, No risk or not sure [25].

Perceived severity/risk of developing severe disease: Refers to a person's subjective perception of the seriousness of contracting COVID-19 disease after infection and is measured as High, Moderate, Low, No risk or not sure [25].

### Data collection instruments

The survey consisted of questions that assessed (1) socio-demographic characteristics; (2) COVID-19 preventive behaviours and perceived risk perception of COVID-19; and (3) intention to receive a COVID-19 vaccine and vaccine efficacy and safety.

### Data processing and analysis

After the responses from the Google form were downloaded in Excel form, the data were checked for completeness and consistency, then compiled and coded. Then, the data were exported to STATA version 14 statistical software for analysis. Frequencies and cross-tabulations were used to summarise descriptive statistics of the data and tables were used for data presentation. Binary logistic regression was employed to identify factors associated with the outcome variable. Those variables with a *P*-value ≤0.2 from the bi-variable analysis were candidates for multivariable analysis. The multivariable analysis was used to declare the significance of the association at a *P*-value of 0.05 was used. Moreover, the association between independent and dependent variables was measured using odds ratios with a 95% CI. Model fitness was checked by using the Hosmer–Lemeshow goodness of fit test (*P*-value = 0.485).

### Data quality assurance

The web-based self-administered questionnaire was pretested by taking 5% of the sample size before the actual data collection time. After the pretest was conducted, amendments to the instrument, like wording and formatting were corrected. The tool was first developed in the English language and was translated into the Amharic language with back translation to English to check its consistency. The data collectors from each hospital were recruited and training was given on the objective of the study, instrument and data collection procedures by the principal investigator. To ensure data quality, the principal investigator reviewed each questionnaire daily and checked for completeness. Cronbach's *α* value was done to check its reliability with an item score of 0.892.

### Ethics approval and consent to participate

This study was approved by the institutional review board of Debre Berhan University *(Protocol no.: P015)*. Respondents were informed that their participation was voluntary, and consent was implied by the completion of the questionnaire. Confidentiality was maintained by avoiding registration of personal identifiers like names on the questionnaire and also, no raw data were given to anyone other than the investigator.

## Result

### Socio-demographic characteristics of participants

Out of 440 HCWs who were invited to the online survey, 418 participants (95%) showed their willingness by filling in the questionnaire. The mean age of the study participants was about 30 years with a standard deviation of 4.10. Of all respondents, 62.4% were male, 85.65% reported that they are Orthodox Christian in religion, 55.3% were married, and around 55% of the participants had a Bachelor of Science (BSc) degree or below education level. Similarly, more than half of the participants (53.4%) reported that they have a household number of two or less and the majority of the participants (85.4%) received 6991 to 12 800 ETB monthly salary. Surprisingly, all the participants reported that they used public transport during the COVID-19 pandemic. Nearly 25% of the participants had a household member 60 years or above, and almost all (99.0%) were not active smokers (Table 1).

**Table 1.** Socio-demographic characteristics of health care workers in Amhara region referral hospitals, Ethiopia, 2021 (*n* = 418)

Variables	Categories	Frequency	Percentage	Vaccine hesitancy	
				Yes (%)	No (%)
Sex	Male	261	62.4	110 (42.1)	151 (57.9)
	Female	157	37.6	82 (52.2)	75 (47.8)
Age in years	≤25 years	49	11.7	28 (57.1)	21 (42.9)
	26–30 years	234	56.0	112 (47.9)	122 (52.1)
	≥31 years	135	32.3	52 (38.5)	83 (61.5)
Religion	Orthodox	358	85.6	164 (45.8)	194 (54.2)
	Muslim	40	9.6	20 (50.0)	20 (50.0)
	Protestant	20	4.8	8 (40.0)	12 (60.0)
Marital status	Single	163	39.0	72 (44.2)	91 (55.8)
	Married	231	55.3	112 (48.5)	119 (51.5)
	Separated/divorced	24	5.7	8 (33.3)	16 (66.7)
Educational level	BSc and below	230	55.0	92 (40.0)	138 (60.0)
	MSc and above	188	45.0	100 (53.2)	88 (46.8)
Household number	≤2 individuals	223	53.4	116 (52.0)	107 (48.0)
	3–4 individuals	128	30.6	44 (34.4)	84 (65.6)
	≥5 individuals	67	16.0	32 (47.8)	35 (52.2)
Monthly income	≤6990	27	6.5	8 (29.6)	19 (70.4)
	6991–12 800	357	85.4	169 (47.3)	188 (52.7)
	≥12 801	34	8.1	15 (44.1)	19 (55.9)
Use public transportation	Yes	418	100.0	192 (45.9)	226 (54.1)
	No	0	0.0	0	0
Has school age child	Yes	132	31.6	36 (27.3)	96 (72.7)
	No	286	68.4	156 (54.5)	130 (45.5)
Household ≥60 years	Yes	103	24.6	48 (46.6)	55 (53.4)
	No	315	75.4	144 (45.7)	171 (54.3)
Active smoker	Yes	4	0.9	0	4 (100.0)
	No	414	99.1	192 (46.4)	222 (53.6)

### Respondents' COVID-19 preventive behaviours, health status and perceived COVID-19 risk

Of all study participants, nearly two-thirds (63.6%) were in compliance with social isolation during the COVID-19 pandemic. Similarly, 71.3% wore masks, almost 57% kept physical distance, and 73.2% of the participants washed their hands with soap at least six times per day during the pandemic. Nearly half (48.3%) of the HCWs undertook the COVID-19 test and 56.2% of the HCWs perceive their health status as very good. Only 3.6% and 3.8% of the HCWs reported that they had any chronic illness or autoimmune disease, respectively. Of all participants, 46.4% reported that they are not confident in the capacity of health services to respond to the COVID-19 pandemic and only 45.7% of the participants reported that the information provided by the public health authorities was clear. Nearly half (52.4%) of the participants considered themselves at high risk of getting COVID-19 infection. Only 21% were confident in the efficacy and safety of the vaccine, and 52.4% of the participants reported that they were not sure whether the side effects of the vaccine were tolerable or not (Table 2).

**Table 2.** Respondents' health status, COVID-19 preventive behaviours and perceived risks in Amhara region referral hospital (*n* = 418)

				Vaccine hesitancy	
Variables	Categories	Frequency	Per cent	Yes (%)	No (%)
Social isolation	Yes	266	63.6	80 (30.1)	186 (69.9)
	No	152	36.4	112 (73.7)	40 (26.3)
Wear mask	Yes	298	71.3	112 (37.6)	186 (62.4)
	No	120	28.7	80 (66.7)	40 (33.3)
Keep physical distancing	Yes	238	56.9	60 (25.2)	178 (74.8)
	No	180	43.1	132 (73.3)	48 (26.7)
Wash hand ≥6 times/day	Yes	306	73.2	112 (36.6)	194 (63.4)
	No	112	26.8	80 (71.4)	32 (28.6)
Suspected COVID-19 diagnosis	Yes	242	57.9	52 (21.5)	190 (78.5)
	No	176	42.1	140 (79.5)	36 (20.5)
Undergone COVID-19 test	Yes	202	48.3	64 (31.7)	138 (68.3)
	No	216	51.7	128 (59.3)	88 (40.7)
Own health status perception	Good	183	43.8	88 (48.1)	95 (51.9)
	Very good	235	56.2	104 (44.3)	131 (55.7)
Any chronic illness	Yes	15	3.6	9 (60.0)	6 (40.0)
	No	403	96.4	183 (45.4)	220 (54.6)
Autoimmune disease	Yes	16	3.8	8 (50.0)	8 (50.0)
	No	402	96.2	184 (45.8)	218 (54.2)
Confidence on health services	Not confident	194	46.4	96 (49.5)	98 (50.5)
	Confident	184	44.0	72 (39.1)	112 (60.9)
	Very confident	40	9.6	24 (60.0)	16 (40.0)
Information by health Authorities	Clear	191	45.7	92 (48.2)	99 (51.8)
	Inconsistent	93	22.2	36 (38.7)	57 (61.3)
	Unclear	134	32.1	64 (47.8)	70 (55.2)
Adequacy of measures by the Ethiopian gov't	Adequate	100	23.9	36 (36.0)	64 (64.0)
	Not adequate	132	31.6	84 (63.6)	48 (36.4)
	Not very adequate	186	44.5	72 (38.7)	114 (61.3)
Risk to get COVID-19 infection	Low risk	96	23.0	56 (58.3)	40 (41.7)
	Moderate risk	103	24.6	56 (54.4)	47 (45.6)
	High risk	219	52.4	80 (36.5)	139 (63.5)
Risk to severe disease after COVID-19 infection	Moderate to high risk	115	27.5	68 (59.1)	47 (40.9)
	Low risk	223	53.4	92 (41.3)	131 (58.7)
	No risk/not sure	80	19.1	32 (40.0)	48 (60.0)
Confident on efficacy and safety of the vaccine	Confident	88	21.1	4 (4.5)	84 (95.5)
	Not confident	100	23.9	96 (96.0)	4 (4.0)
	Not very confident	230	55.0	92 (40.0)	138 (60.0)
Side effects of the vaccine	Tolerable	140	33.5	44 (31.4)	96 (68.6)
	Not tolerable	59	14.1	28 (47.5)	31 (52.5)
	Not sure	219	52.4	120 (54.8)	99 (45.2)

### Determinants of COVID-19 vaccine hesitancy among health care workers

Overall, 45.9% (*n* = 192) of study participants reported that they will hesitate or refuse to take the COVID-19 vaccine by the time they get the chance to be vaccinated (95% CI 41.2–50.8).

In order to identify factors that could determine vaccine hesitancy among HCWs, both bi-variable and multi-variable binary logistic regression models were applied. After applying bi-variable binary logistic regression, variables with a *P*-value of 0.2 or lower were taken to multi-variable binary logistic regression. Then, age ≤25 years (adjusted odds ratio (aOR) 5.8 (95% CI 1.6–12.5)), do not wear a mask during the pandemic (aOR 2.4 (95% CI 1.04–5.3)), do not comply with physical distancing during the COVID-19 pandemic (aOR 3.6 (95% CI 1.7–7.9)), unclear information provided by public health authorities (aOR 2.5 (95% CI 1.3–5.0)), low risk of getting COVID-19 infection (aOR 2.8 (95% CI 1.4–5.5)) and not sure regarding the tolerability of side effects of the vaccine (aOR 3.8 (95% CI 2.0–7.1)) were found to be significant predictors of hesitancy towards COVID-19 vaccine at a *P*-value <0.05 (Table 3).

**Table 3.** Bi-variable and multivariable binary logistic regression analysis of factors associated with vaccine hesitancy among HCWs (*n* = 418)

Variables	Categories	Vaccine hesitancy		COR (95% CI)	AOR (95% CI)
		Yes	No		
Sex	Male	110	151	1.0	
	Female	82	75	1.5 (1.01–2.2)*	1.6 (0.8–3.1)
Age in years	≤25 years	28	21	2.1 (1.1–4.1)*	5.6 (1.6–12.5)**
	26–30 years	112	122	1.5 (0.9–2.3)*	1.2 (0.6–2.3)
	≥31 years	52	83	1.0	
Marital status	Married	112	119	1.0	
	Unmarried	72	91	0.8 (0.6–1.3)	0.52 (0.3–1.0)
	Divorced/separated	8	16	0.5 (0.2–1.3)*	0.4 (0.1–1.3)
Educational level	BSc and below	92	138	1.0	
	MSc and above	100	88	1.7 (1.2–2.5)*	1.2 (0.7–2.3)
Household number	≤2 individuals	116	107	1.2 (0.7–2.1)	0.7 (0.2–2.3)
	3–4 individuals	44	84	0.6 (0.3–1.5)*	0.5 (0.2–1.2)
	≥5 individuals	32	35	1.0	
Monthly income	≤6990 ETB	8	19	1.0	
	6991–12 800 ETB	169	188	2.1 (0.9–5.0)*	3.3 (0.9–11.6)
	≥12 801 ETB	15	19	1.9 (0.6–5.5)	2.7 (0.5–13.3)
Has school age child	Yes	36	96	1.0	
	No	156	130	3.2 (2.0–5.0)*	2.5 (0.9–6.4)
Social isolation	Yes	80	186	1.0	
	No	112	40	6.5 (4.2–10.2)*	1.2 (0.5–2.7)
Wear mask	Yes	112	186	1.0	
	No	80	40	3.3 (2.1–5.2)*	2.4 (1.04–5.3)**
Wash hands	Yes	112	194	1.0	
	No	80	32	4.3 (2.7–6.9)*	0.9 (0.4–2.2)
Physical distancing	Yes	60	178	1.0	
	No	132	48	8.2 (5.3–12.7)*	3.6 (1.7–7.9)**
Undergone COVID test	Yes	64	138	1.0	
	No	128	88	3.1 (2.1–4.7)*	1.6 (0.9–3.0)
Confidence on health services	Not confident	96	98	0.7 (0.3–1.3)	0.7 (0.3–2.0)
	Confident	72	112	0.4 (0.2–0.9)*	1.3 (0.5–3.5)
	Very confident	24	16	1.0	
Information by health authorities	Clear	92	99	1.0	
	Inconsistence	36	57	0.7 (0.4–1.1)*	0.8 (0.4–1.7)
	Unclear	64	70	0.9 (0.6–1.5)	2.5 (1.3–5.0)**
Measures by the gov't	Adequate	36	64	1.0	
	Not adequate	84	48	3.1 (1.8–5.3)*	1.5 (0.7–3.4)
	Not very adequate	72	114	1.1 (0.7–1.9)	0.8 (0.4–1.8)
Risk to get COVID-19 infection	Low risk	56	40	2.4 (1.5–3.9)*	2.8 (1.4–5.5)**
	Moderate risk	56	47	2.1 (1.3–3.3)*	1.7 (0.9–3.4)
	High risk	80	139	1.0	
Risk to sever disease	Moderate to high	68	47	2.2 (1.2–3.9)*	0.9 (0.4–2.3)
	Low risk	92	131	1.1 (0.6–1.8)	0.8 (0.4–1.8)
	No risk/not sure	32	48	1.0	
Side effects of COVID-19 vaccine	Tolerable	44	96	1.0	
	Not tolerable	28	31	1.9 (1.1–3.7)*	1.7 (0.7–3.9)
	Not sure	120	99	2.6 (1.7–4.1)*	3.8 (2.0–7.1)**

AOR, adjusted odds ratio; BSc, Bachelor of Science; COR, crude odds ratio; ETB, Ethiopian Birr; MSc, Master of Science.

*Significant at *P*-value ≤0.2; **significant at *P*-value <0.05.

## Discussion

In the current study, the overall magnitude of vaccine hesitancy towards COVID-19 among HCWs was 45.9% (95% CI 41.2–50.8). This is in line with a study conducted in Saudi Arabia and London [10, 16]. The magnitude of vaccine hesitancy among HCWs in the current study is higher than in a study conducted at a worldwide level, in Germany, in Vietnam, in Southern Italy, and in a survey in France and French-speaking parts of Belgium and Canada [3, 5, 11, 12, 15]. But, it is lower than studies conducted in the Democratic Republic of the Congo and A Large-Scale Multinational Study conducted among Arab HCWs [6, 17]. This discrepancy might be due to differences in socio-demographic characteristics, differences in religious beliefs regarding the vaccine and time of study conducted. Additionally, decreased case reports in Ethiopia as compared to other European countries might have contributed to increased hesitancy for COVID-19 vaccination in the current study.

This study revealed that the likelihood of vaccine hesitancy among HCWs age ≤25 years was 5.6 times greater than among HCWs categorised at age ≥31 years (aOR 5.6 (95% CI 1.6–12.5)). This is in line with the studies conducted in Ethiopia, London and Greece [4, 10, 13, 26]. This increased COVID-19 hesitancy among younger ages (≤5 years) might be due to WHO's declaration regarding high-risk groups for COVID-19 infection and death. The WHO has declared that the COVID-19 pandemic is more prevalent and more severe among those with advanced age than younger ones. This leads to younger participants hesitating more towards receiving the vaccine than older participants [1]. Hence, this finding indicates that younger age HCWs should be a target population to change their perception of COVID-19 infection because they are more hesitant to get vaccinated.

The current study showed that HCWs who did not wear masks every time they left home during the COVID-19 pandemic were 2.4 times more reluctant to take the COVID-19 vaccine (aOR 2.4 (95% CI 1.04–5.3)) compared to HCWs who wore masks. Similarly, the odds of vaccine hesitancy was 3.6 times higher among HCWs who did not comply with physical distancing during the COVID-19 pandemic than among participants who were fully compliant with physical distancing (aOR 3.6 (95% CI 1.7–7.9)). This positive relationship between vaccine hesitancy with those who did not wear masks and did not comply with physical distancing during the pandemic might probably be due to a negative attitude towards COVID-19 and its preventive measures. Participants who did not comply with COVID-19 preventive measures during the pandemic are more likely to believe in conspiracy theories such as COVID-19 is not real or does not exist. Others might think that even if it existed, it could not be a serious disease. Additionally, some of the participants might be concerned about vaccines' incompatibility with religious beliefs [5, 27].

Information provided by public health authorities regarding COVID-19 showed a significant association with hesitancy towards COVID-19 vaccination. HCWs who perceived the information provided by public health authorities as unclear were 2.5 times more likely to hesitate against receiving the COVID-19 vaccine as compared to those who perceive the information as clear (aOR 2.5 (95% CI 1.3–5.0)). This finding is in line with studies conducted in Egypt and Greece [8, 13]. Trust in the public health authorities through which information about vaccines is provided is an essential drive for vaccine acceptance among people, including HCWs. So, HCWs who perceive the information provided by public health authorities is unclear might be prone to mistrust in public health authorities to ensure vaccine safety, lack of trust in the manufacturing and country of production of vaccines, vaccine technology, and display a lack of confidence in the government. These situations in turn result in hesitancy towards receiving the vaccine for COVID-19 [3, 5, 27, 28]. Thus, the provision of clear and consistent information about the COVID-19 infection and the vaccine, both for the general population and even for HCWs, is crucial to combat vaccine hesitancy.

Likewise, HCWs' perception regarding their risk of getting COVID-19 infection was found to be significantly associated with COVID-19 vaccine hesitancy. The odds of vaccine hesitancy was 2.8 times higher among HCWs who perceive themselves as low risk of getting COVID-19 infection as compared to those who perceive themselves as high risk (aOR 2.8 (95% CI 1.4–5.5)). This is congruent with studies conducted in Vietnam and Southern Italy [12, 15]. HCWs who perceive themselves as being at a lower risk are not highly concerned about getting COVID infection. Consequently, they will not be fully engaged in preventive measures for COVID-19, including vaccination [16].

Lastly, HCWs who were not sure regarding the tolerability of the vaccine side effects were 3.8 times more hesitant to take the COVID-19 vaccine compared to HCWs who thought that the side effects of the vaccine were tolerable (aOR 3.8 (95% CI 2.0–7.1)). This is in line with a study conducted in Egyptian HCWs [8]. This is because hesitancy is mostly driven by concerns about vaccine safety, efficacy and side effects. These are also the top three major reasons for COVID-19 vaccination hesitancy among HCWs worldwide. This means, HCWs who are not sure whether the side effects of the COVID-19 vaccine are tolerable or not, are highly reluctant to take the vaccine [3, 5, 6]. Hence, the provision of clear and scientifically approved information about COVID-19 vaccine safety and associated adverse or side effects for HCWs should be a priority activity by public health authorities.

## Conclusion

A considerable proportion of HCWs were found hesitant towards receiving the COVID-19 vaccine once it is available. Being younger age, non-compliance with COVID-19 infection preventive measures, hearing unclear COVID-19-related information from health authorities, considering oneself at low risk of getting COVID-19 infection and not being sure regarding tolerable side effects of the vaccine were statistically significant factors that determined the magnitude of vaccine hesitancy. So, in order to mitigate this challenge, health authorities should provide brief information to the HCWs who are the role models for the general population.

## Availability of data and materials

The datasets used during the current study are available from the corresponding author.
